# Man an Object of Zoology

**Published:** 1887-10

**Authors:** S. W. Stiles

**Affiliations:** Georgia


### MAN AN OBJECT OF ZOOLOGY.

BY S. W. STILES, M. D., GA.

[*Continued from page 359.*]

We will now proceed to speak of the different races and types of man in different countries, and as Adam was the crowning point of creation made like unto God, in his own image, we shall, for a few chapters, pay particular attention to him alone.

There are no essential differences between the various races of the human species in the execution of the animal functions. The general law that animals produce their like, by which uniformity of species is maintained, suffers some exceptions. Children do not always resemble their parents. Among the Otaheitans, descended from the Malay race, individuals with light-brown or sandy hair and fair complexions are not uncommon, and red haired persons have been observed in most of the dark nations, as the Eskimaux, Islanders of New Guinea and New Zeland, and the Negroes. The origin of Albinos, particularly in the dark races, is a remarkable example of native variety of color. In the mixed breeds, although the children generally partake of the character of both parents, they sometimes resemble one only. A negress had twins by an Englishman: one was perfectly black, with short, woolly hair, the other was light, with long hair. Variations of color analogous to those just enumeratod are of daily occurrence among animals as in the production of black sheep, cats, horses, foxes, etc. White sheep may produce black lambs, and gray rabbits may bring forth either white or black ones. Examples occur of individuals spotted with different colors, but they are by no means so common, as those of spotted animals.

Persons of the black race are sometimes marked with patches of white of various sizes and number. This circumstance has led the French writers to call them *negres pies*, or piebald negroes. Blumenbach has described a man of this kind. A young man, perfectly black, excepting the umbilical, and hypogastric regions of the abdomen, and the middle of the lower limbs, including the knees, thighs and legs, which were of a snowy whiteness, but spotted with black, like the skin of a panther; his hair was of two colors: on the middle of the front of the head, from the vertex to the forehead where it ended in a sharp point, there was a white spot with a yellower tinge than those on the legs and thighs, the rest of the hair was jet black, short and woolly. Both the parents of this man were entirely black. These spots, in which the epidermis is perfectly healthy, and which are distinguishable from the rest of the skin, only by their whiteness are not to be confounded with diseases of the organ where the cuticle becomes scaly, which is frequent in some of the black races. The skin differs in some other properties besides its color; it is remarkable for its softness and smoothness in the Carib, Negro, Otaheiteans and Turk. In some of the races it secretes a matter of peculiar odor. The Peruvian Indians, says Humbolt, who in the middle of the night distinguish the different races by their sense of smell, have formed three words to express the odor of the European, the American Indian and the Negro, they call the first *pezun*, the second *poseo*, and the third *graio*.

[To be Continued.]

